# pH Modulation of Efflux Pump Activity of Multi-Drug Resistant *Escherichia coli*: Protection During Its Passage and Eventual Colonization of the Colon

**DOI:** 10.1371/journal.pone.0006656

**Published:** 2009-08-17

**Authors:** Ana Martins, Gabriella Spengler, Liliana Rodrigues, Miguel Viveiros, Jorge Ramos, Marta Martins, Isabel Couto, Séamus Fanning, Jean-Marie Pagès, Jean Michel Bolla, Joseph Molnar, Leonard Amaral

**Affiliations:** 1 Unit of Mycobacteriology, Universidade Nova de Lisboa (IHMT/UNL), Lisboa, Portugal; 2 UPMM, Instituto de Higiene e Medicina Tropical, Universidade Nova de Lisboa (IHMT/UNL), Lisboa, Portugal; 3 Cost Action BM0701 ATENS, Faculdade de Ciências e Tecnologia, Universidade Nova de Lisboa, Caparica, Portugal; 4 Centro de Recursos Microbiológicos (CREM), Faculdade de Ciências e Tecnologia, Universidade Nova de Lisboa, Caparica, Portugal; 5 Centre for Food Safety, School of Agriculture, Food Science and Veterinary Medicine, University College Dublin, Belfield, Dublin, Ireland; 6 UMR-MD-1, IFR88, Facultés de Médecine et de Pharmacie, Université de la Méditerranée, Marseille, France; 7 Medical Microbiology & Immunology, Institute of Medical Microbiology, University of Szeged, Szeged, Hungary; University of Wisconsin-Milwaukee, United States of America

## Abstract

**Background:**

Resistance Nodulation Division (RND) efflux pumps of *Escherichia coli* extrude antibiotics and toxic substances before they reach their intended targets. Whereas these pumps obtain their energy directly from the proton motive force (PMF), ATP-Binding Cassette (ABC) transporters, which can also extrude antibiotics, obtain energy from the hydrolysis of ATP. Because *E. coli* must pass through two pH distinct environments of the gastrointestinal system of the host, it must be able to extrude toxic agents at very acidic and at near neutral pH (bile salts in duodenum and colon for example). The herein described study examines the effect of pH on the extrusion of ethidium bromide (EB).

**Methodology/Principal Findings:**

*E. coli* AG100 and its tetracycline induced progeny AG100_TET_ that over-expresses the *acrAB* efflux pump were evaluated for their ability to extrude EB at pH 5 and 8, by our recently developed semi-automated fluorometric method. At pH 5 the organism extrudes EB without the need for metabolic energy (glucose), whereas at pH 8 extrusion of EB is dependent upon metabolic energy. Phe-Arg β-naphtylamide (PAβN), a commonly assumed inhibitor of RND efflux pumps has no effect on the extrusion of EB as others claim. However, it does cause accumulation of EB. Competition between EB and PAβN was demonstrated and suggested that PAβN was preferentially extruded. A K_m_ representing competition between PAβN and EB has been calculated.

**Conclusions/Significance:**

The results suggest that *E. coli* has two general efflux systems (not to be confused with a distinct efflux pump) that are activated at low and high pH, respectively, and that the one at high pH is probably a putative ABC transporter coded by *msbA*, which has significant homology to the ABC transporter coded by *efrAB* of *Enterococcus faecalis*, an organism that faces similar challenges as it makes its way through the toxic intestinal system of the host.

## Introduction

Bacteria that are orally ingested need to survive when exposed to noxious agents such as toxic bile salts that are present along the digestive tract (ex: duodenum and colon). Resistance of Gram-negative bacteria to bile salts has been attributed to the presence of an efflux pump, which extrudes the agent prior to its reaching sensitive and lethal targets within the bacterium [1]-[4]. With respect to *E. coli*, extrusion of noxious agents is performed primarily by the AcrAB efflux pump, although other efflux pumps may assist in the extrusion process [5]. Collectively, these efflux pumps are classified as belonging to the Resistance Nodulation Division (RND) family of transporters [6], [7]. RND efflux pumps are tripartite units that consist of a TolC protein connected to the transporter protein and provides the conduit for the passage of the exported agent to the outside of the bacterium [8]. The transporter component of the pump is anchored to the plasma membrane of the bacterium by two flanking fusion proteins. The tripartite pump obtains the necessary energy to power extrusion of the agent directly from the trans-membrane proton gradient [9], [10]. This gradient provides the protons that are present in the periplasmic space and, when they enter the transporter at its plasma membrane base, they energize the pump and the agent which is believed to be concentrated within the outer leaflet of the inner membrane is in turn extruded [11]. The proton is then released to the medial side of the plasma membrane. The fusion proteins are believed to physically assist the extrusion of the agent [11].

The proton motive force (PMF) is in part established by protons generated following the hydrolysis of ATP catalyzed by membrane bound ATPases [12]–[16] and by oxidative metabolism [17]. The generation of these protons takes place at sites medial to the inner membrane of the Gram-negative bacterium. After the protons are exported to the periplasm by a variety of transport processes, most of which are then exported to the surface of the cell, the distribution of protons between the periplasm and the cell surface results in a proton gradient that is greatest at the surface of the cell and least in its periplasm. This distribution establishes a relative negative charged periplasmic space and positive charged surface of the cell and results in an electrochemical gradient. The resulting trans-membrane difference in the electrochemical potential of hydrogen ions was at first seen to be the driving force behind the energy consuming enzymes and ATP-synthase and was defined as the proton motive force (PMF) [18]. However, because of the largesse of the bulk water phase the dissemination of protons from the surface of the cell would quickly eliminate the pH gradient across the cell envelope, and hence, the PMF and the energy it provides for driving efflux pumps would be eliminated [18]. Because the PMF is maintained by the bacterium when challenged by changes in the pH of the environment [19], [20] the chemiosmotic theory required revision, and the concept was extended to include the distribution of protons on the surface of the cell which due to the components of the outer cell envelope, would be selectively concentrated and result in a pH of the medium immediately surrounding the surface of the cell that would be much lower than the pH of the bulk medium [21]. This surface distribution of protons therefore assists the bacterial cell in maintaining a PMF that would not be significantly affected by the pH of the medium [22]. Consequently, it is widely held that the pH of the medium should not affect the activity of a RND efflux pump even though there is no evidence in support of this conclusion. Exposure of *Salmonella* to low pH is well known to activate the PmrA/PmrB two component regulatory system which not only allows the organism to survive the low pH of the phagolysosome, but also increases its resistance to antibiotics [23]. Exposure of *E. coli* to low pH activates a wide spectrum of genes, some of which code for cell envelope proteins [24], [25]. These studies suggest that low pH readily activates genes of a Gram-negative bacterium and render the organism resistant. Although the question of whether pH-induced resistance of a Gram-negative involves the activation of genes that regulate and code for efflux pump constituents remains our goal, we have asked the question of whether pH has a direct effect on the RND efflux pump AcrAB of *E. coli*. We herein report that pH significantly modulates the efflux of the known efflux pump substrate ethidium bromide (EB) and correlate this modulation to the challenges that the organism faces when it passes through regions of the intestinal system that differ significantly in pH and yet present the same toxic challenges.

## Results

Because pH affects many activities of the cell envelope surface that affect the growth of *E. coli* [26], [27], the effect of pH on the growth of the *E. coli* AG100 and AG100_TET_ was determined. Briefly, as evident from **Table 1**, the calculated slopes depicting rate of growth, indicate that both strains grow more slowly at pH 5 than at pH 7. However, the rate of growth is twice as slow for the AG100_TET_ as opposed to that of the parental strain AG100. Nevertheless, whereas movement through the low acid components of the digestive tract does not significantly effect the survival of the organism, the presence of toxic agents such as bile salts in the small and large intestine, poses problems of survival if these agents permeate into the bacterium.

**Table 1:** The growth rates of *E. coli* AG100 and AG100_TET_ in MH at pH 5, 7 and 8.

*E. coli* strain	Slope at pH 5	Slope at pH 7	Slope at pH 8
**AG100**	0.22	0.40	0.29
**AG100_TET_**	0.06	0.22	0.19

**Legend**: Growth rates are presented as slopes which are calculated from the number of hours of culture divided into the total optical density (OD at 600 nm) of the culture. The steeper the slope the faster the growth rate.

**Figure 1:**
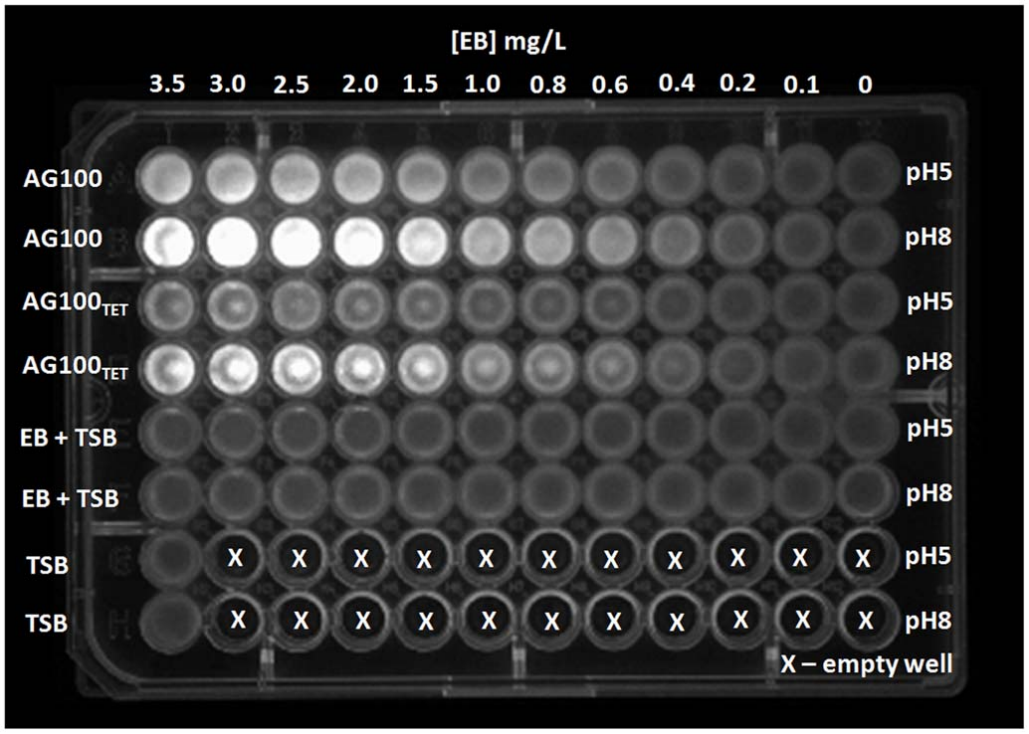
Fluorescence of the strains *E. coli* AG100 and AG100_TET_ in EB containing media at pH 5 and pH 8. From the left to the right, the fluorescence emitted by the bacteria grown in decreasing concentrations of EB during 16 hours. The controls of the media (TSB with or without EB) at pH 5 and pH 8 are presented in the last four rows of the plate.

The ability of each strain to extrude different concentrations of EB were verified by using a 96-well microplate containing Tryptic Soy broth (TSB) with increasing concentrations of EB as described by Figure 1. The assay, performed at pH 5 and 8, indicates that AG100_TET_ begins to fluoresce in medium at pH 8 at a concentration of 0.6 mg/L of EB, whereas the wild-type AG100 begins to fluoresce at 0.2 mg/L of EB. This difference is in accordance to the degree of AcrAB efflux pump expression, namely the AG100_TET_ over-expresses this pump 6-fold over that of AG100, as shown by real-time quantitative reverse transcriptase-PCR (qRT-PCR) [5]. At the lower pH, the strains require higher concentrations of EB to fluoresce: 1.5 and 0.6 mg/L for the AG100_TET_ and AG100, respectively.

**Figure 2:**
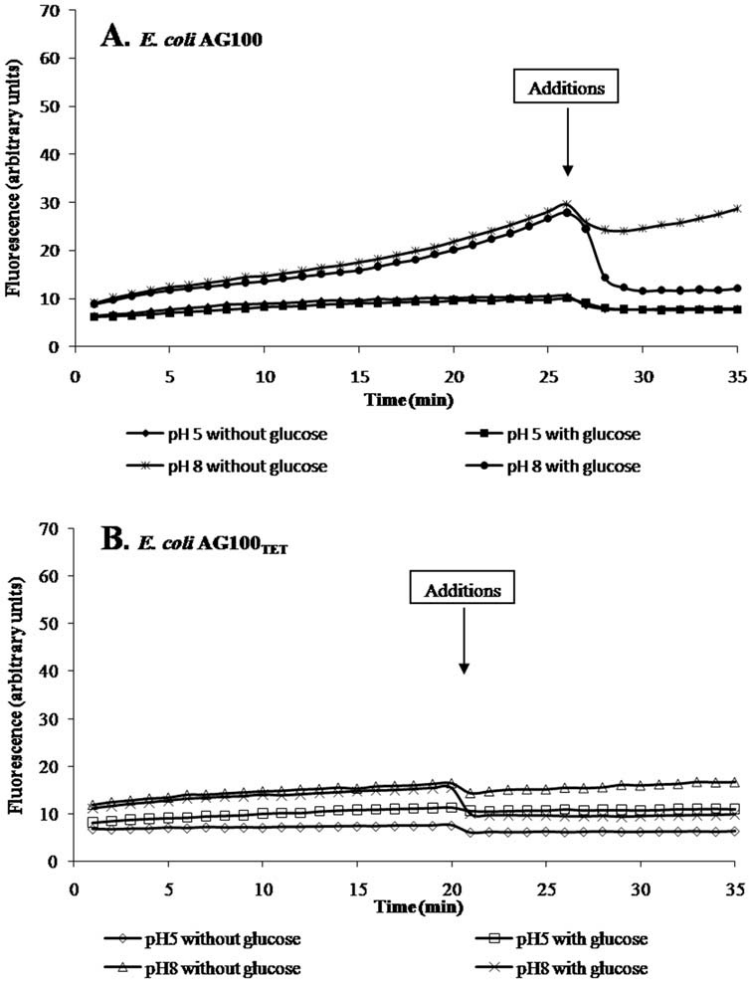
The effect of pH and the need for metabolic energy for efflux of EB by *E. coli* AG100 (Fig. 2A) and AG100_TET_ (Fig. 2B). Accumulation of EB in glucose-free saline at pHV5 and 8 for 25 minutes. Instrument is stopped and glucose-free and glucose-saline pHV5 and 8, respectively, is added and the instrument restarted. A slight drop of recording takes place when the instrument is stopped and when restarted measurement of fluorescence continues. Note. Whereas the addition of glucose-saline at pH 8 to the tube containing glucose-free saline at pH 8 causes an immediate drop of fluorescence, the addition of glucose-free medium does not, and the slope representing the increase of fluorescence remains equal to that exhibited during accumulation in glucose-free saline at pH 8. Accumulation of EB in glucose-free saline at pH 5 is minimal (just above background) and remains unaltered with the addition of either glucose-free or glucose-saline at pH 5.

Accumulation of EB by AG100 in glucose-free saline and the effect of pH are shown in **Figure 2A**. Whereas no significant accumulation of EB takes place in glucose-free saline pH 5, accumulation of EB in glucose-free saline at pH 8 takes place during the first 25 minutes. The need for metabolic energy at pH 8 for efflux of EB is illustrated with the addition of glucose to the cells after 25 minutes of accumulation of EB in glucose-free saline at pH 8, inasmuch as the amount of fluorescence drops and is maintained at the initial level noted at the beginning of the accumulation period. The addition of glucose-free saline at pH 8 does not affect the rate of EB accumulation noted during the accumulation period of the assay. These results clearly demonstrate the activity of the intrinsic efflux pump system of the wild-type *E. coli* AG100 strain that is operating at pH 8. At pH 5, it is assumed, at this time, that the reason for little or no accumulation of EB regardless of metabolic energy (glucose) is due to the activity of the PMF dependent RND efflux pump.

Accumulation and efflux of EB by the AG100_TET_ strain, that over-expresses the AcrAB efflux pump, compared to AG100 [5], [25], and the need for metabolic energy for efflux at pH 8 and not at pH 5 is similarly demonstrated by **Figure 2B**.

**Figure 3:**
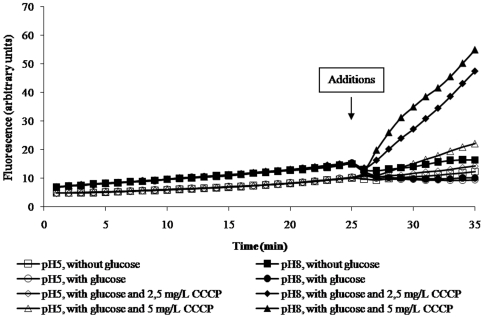
Effect of CCCP concentrations on efflux of EB by *E. coli* AG100_TET_ at pH 5 and 8. The same protocol of accumulation was followed as for Figure 2. After 25 minutes, saline with glucose and CCCP was added to the tubes. The control without CCCP and without glucose is also presented.

The demonstration of an RND type efflux pump of a Gram-negative is usually conducted with the PMF un-coupler carbonyl cyanide *m*-chlorophenylhydrazone (CCCP) at pH 7 and in the absence of metabolic energy. Given the demonstration that at pH 8 metabolic energy optimizes efflux, the activity of varying concentrations of this agent at pH 5 and 8 on the efflux of EB after the fluorochrome has accumulated in the absence of glucose has been studied and the results obtained described by **Figure 3** for the AG100_TET_ that over-expresses its AcrAB efflux pump [5], [25]. At pH 5 and 8, CCCP immediately prevents efflux and increases the rate and extent of accumulation of EB in a concentration dependent manner. However, comparison of the slopes of accumulation between that at pH 5 and pH 8 indicates that at pH 8 the effect of CCCP is considerably greater. Because these assays were conducted at the same time and under the same conditions other than pH, the lesser effects of CCCP at pH 5 may be due to the large contribution of protons at pH 5 that exceeds the proton binding capacity of the CCCP concentration. At pH 8, due to a far lower concentration of available protons, the concentration of CCCP essentially binds all of the protons and thereby it completely inhibits efflux of EB. The same effects were produced by CCCP and its modulation of EB efflux by the intrinsic efflux pump system of the AG100 wild-type strain (data not shown). The observation that CCCP promotes the retention of EB at pH 5 demonstrates that the much lower accumulation of EB at this pH is due to efflux as opposed to a decrease of permeability to EB.

**Figure 4:**
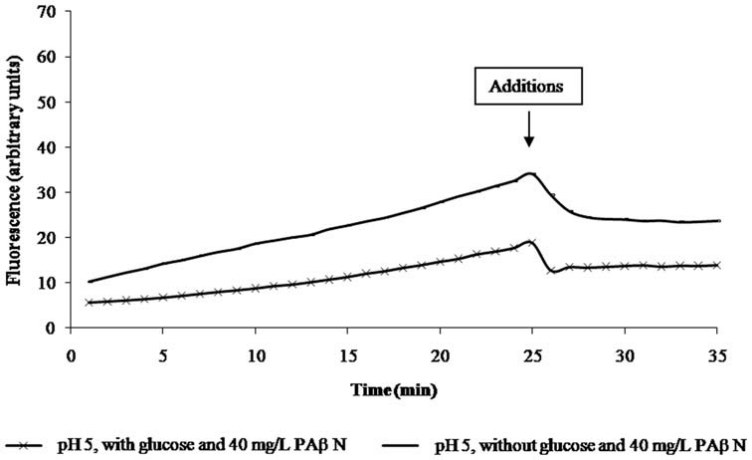
Effects of PAβN on efflux of EB by AG100_TET_ at pH 5 and 8. The same protocol of accumulation was followed as for figure 2. After 25 minutes media with glucose and PAβN was added to the tubes.

**Figure 5:**
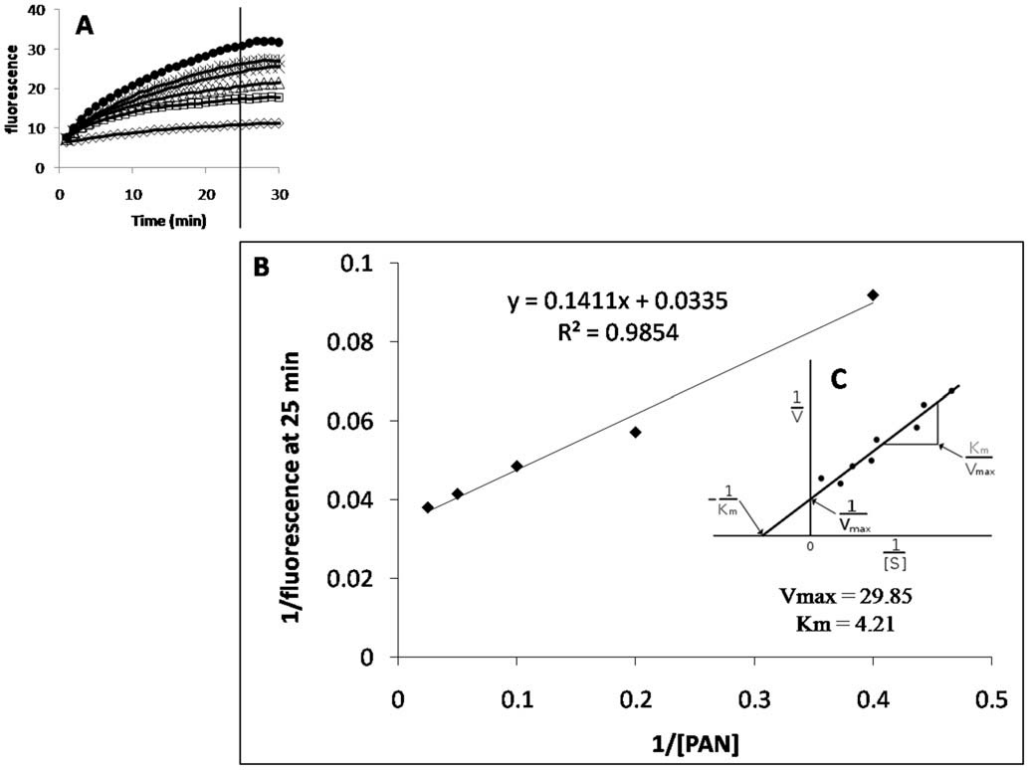
Competition between EB and PAβN: calculation of K_m_ for PAβN relative to EB. Increasing concentrations of PAβN from 1 to 40 mg/L caused increase of fluorescence (A). This data was then used for the derivation of the PAβN K_m_ initially plotted by (B) and data employed in the Michaelis-Menten (C).

Phe-Arg β-naphtylamide (PAβN) has been used to demonstrate the presence of an active efflux pump that renders some Gram-negative bacteria resistant to given antibiotics. This demonstration involves the reduction of a minimum inhibitory concentration (MIC) of a given antibiotic over a 16 hour culture. However, our previous studies suggested that PAβN is not an inhibitor of an efflux pump but rather a competitor of other efflux pump substrates for extrusion [25], a suggestion also made by others [26]. This postulated preferential extrusion of PAβN is believed to result in the increased concentration of the antibiotic which eventually reaches a level that inhibits the replication of the organism. Moreover, if PAβN is an inhibitor of an RND efflux pump, then it should inhibit the efflux of EB at pH 5 inasmuch as the efflux of EB at pH 5 is independent of metabolic energy and dependent upon the PMF. The effect of PAβN on the accumulation and efflux of EB by the AG100_TET_ at pH 5 is described by **Figure 4**. As evident from this figure, the addition of PAβN has no effect on the efflux of EB at either pH. However, because PAβN competes with EB, as the concentration of PAβN is increased, more and more EB would be expected to accumulate. This anticipated relationship was exploited for the derivation of a K_m_ for PAβN relative to EB at pH 5 inasmuch as at this pH metabolic energy is not needed and PAβN has no effect on efflux of EB. Moreover, the dissociation constant of EB from the AcrB transporter is lowest at pH 5 [28], a condition that is necessary for continuous efflux of EB. As described by the composite **Figure 5**, as the concentration of PAβN is increased from 1 to 40 mg/L, the amount of EB accumulation is proportionately increased. Employing Michaelis-Menten formulae, the K_m_ for PAβN representing competition between PAβN and EB was calculated to be 4.21 mg/L.

**Figure 6:**
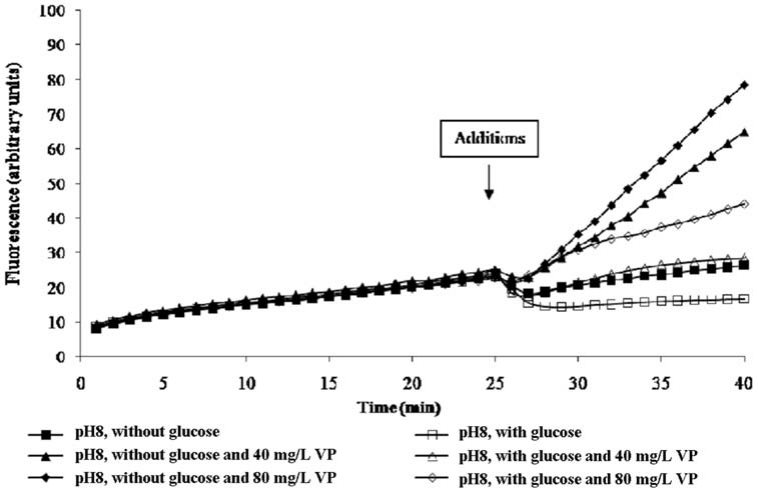
The effects of concentrations of verapamil (VP) on the efflux of EB by AG100 at pH 8.

Verapamil inhibits ABC transporters of *Staphylococcus aureus* [29] and mycobacteria [30]. However, there is little information regarding the effects of verapamil on efflux activity of a Gram-negative such as *E. coli*. Considering the possibility that the study of agents for inhibitory activity against efflux pumps is always conducted at neutral or near neutral, and because at pH 8 efflux of EB by the *E. coli* strains employed in this study is dependent upon metabolic energy, suggesting the involvement of an ABC type transporter, we have evaluated the effects of concentrations of verapamil on efflux of EB. As evident by **Figure 6**, and consistent with previously presented data, at pH 8 efflux of EB is dependent upon the presence of metabolic energy. The addition of verapamil in the absence of metabolic energy, promotes a concentration dependent inhibition of efflux which causes proportional increases in accumulation of EB. The inhibitory effect on efflux by verapamil is significantly decreased when metabolic energy is present. These results suggest that at pH 8, efflux of EB is at the very least, partially conducted by an ABC type transporter. Similar results were obtained with the AG100_TET_ strain (data not shown).

## Discussion

The results presented in this study demonstrate that the pH of the milieu modulates the over-all activity of the intrinsic and over-expressed efflux pump system of *E. coli*. pH modulation of genes that code for ion transporters of *E. coli* has been previously demonstrated by others [31]. However, this is the first time that pH is shown to modulate the accumulation and extrusion of an efflux pump substrate such as EB.

The effect of pH on the cell envelope, its constituents, genes that regulate growth and metabolism has been reported and reviewed in detail [31]. As also shown in the current study, low and high pH reduces optimum growth demonstrable at pH 7. However, low pH is not a problem for *E. coli* inasmuch as its survival, regardless of a slower growth rate, is not significantly affected as evident from the success of an orally consumed *E. coli* reaching and successfully colonizing the colon. The survival of the organism is dependent upon its ability to extrude noxious agents present in the digestive system of the host. Clearly, the ability to extrude a noxious agent is intrinsically present in wild-type *E. coli*. The extrusion capability when over-expressed, make therapy of multi-drug resistant (MDR) *E. coli* infections problematic.

The selection of pH affords the distinction between an efflux pump system immediately dependent on the PMF, such as a RND efflux pump, and one that is dependent upon metabolic energy and inhibited by verapamil. Because the *msbA* gene of *E. coli* is similar to the ABC transporter gene *efrAB* of *E. faecalis* [32], [33], we believe that the glucose dependent efflux at pH 8 noted in our study may be conducted by the putative MsbA transporter. Further studies have to be performed in order to identify the ABC transporter in this study. Nevertheless, an ABC type transporter system that has the capability to extrude the efflux pump substrate EB has now been demonstrated for the first time in *E. coli*.

The current study utilizes a modification of the semi-automated fluorometric method previously described [34]. Firstly, whereas in the initial method a pH of 7 was maintained for the entire study, the current method utilizes a range of pH and glucose-free saline for the EB accumulation phase. Secondly, whereas the initial method employed a wash for the removal of EB, the medium replaced with EB free-saline, and the efflux of EB was then followed, in the current method after the accumulation phase, glucose-saline of matching pH is added and, in accordance to the criteria governing the source of energy driving the pump, efflux of EB is followed. This simple change in the method makes it far less cumbersome, eliminates the possibility of leakage of EB due to the washing procedures and the large variation between replicates due to the wash, and perhaps of greatest importance, the rate of efflux, almost from the very beginning can be noted. We say “almost” because the stopping of the instrument after the EB accumulation phase, the additions and their number, the return of the samples to the instrument and restarting the instrument introduce a variable of time during which the addition of glucose is immediately followed by efflux of EB. However, in our hands, this time variable did not exceed 2.5 minutes when as many as 12 individual additions were made, and did not produce any qualitative variation between replicate runs.

Our results also indicate that the widely used efflux pump substrate PAβN is not an inhibitor of the efflux pump as previously suggested by others [26], and that the initial evidence provided in support of this contention [32] has now been verified by the current study. Furthermore, the degree of competition between PAβN and EB for extrusion by wild-type *E. coli* AG100 has been demonstrated and has resulted in a K_m_ for PAβN relative to EB of 4.21 mg/L.

Our study supports the contention that the survival of a bacterium as it makes its way through the digestive system and eventual colonization of the colon, is assured by the activity of two types of efflux pump systems: one that operates directly from PMF derived energy and the other from a putative ATP binding transporter. It is supposed that when the bacterium reaches the duodenum, the toxicity presented by high concentrations of bile salts must be obviated and this is accomplished by the extrusion of these toxic substances by an RND efflux pump such as the AcrAB of *E. coli*. Because our study demonstrates that the intrinsic efflux pump system of wild-type *E. coli* is perfectly capable of extruding EB in glucose-free saline at pH 5, and this extrusion can be eliminated by a un-coupler of the PMF, it should be this PMF dependent pump system which protects the bacterium while passing through the duodenum. There is no need for an over-expressed PMF dependent efflux pump system. When the organism reaches the colon, bile salts, as well as many secreted agents produced by the normal flora, are present and are toxic to the organism. The pH of the colon is near 7 and because of the scarcity of protons in this bulk medium the concentration of surface bound protons must be affected, and is probably much lower than that at pH 5. If the RND pump is to operate under these conditions, the PMF must be maintained and the protons needed to activate the RND efflux pump must be available in the periplasm [11]. Subsequent to the extrusion of the noxious agent the protons are released to the cytoplasm. Two general situations are expected from the activity of the RND efflux pump at pH 7 or higher: firstly, the consumption of protons from the periplasm reduces the PMF unless protons are replaced from the surface of the cell. The process of replacement is probably limited and inadequate for the maintenance of an RND efflux system under these conditions; secondly, the release of protons to the medial side of the cytoplasm membrane will decrease the pH and the synthesis of ATP by ATP-synthase is favoured [34], [35]. The utilization of protons for the synthesis of ATP insures that the pH gradient between the periplasm and cytoplasmic side of the plasma membrane is maintained. The ATP generated is then bound by the ABC transporter, hydrolyzed and the energy from this process activates the ABC transporter to extrude the noxious agent. Metabolic energy also contributes to this process. In the toxic environment of the colon, we suppose the organism receives protection from the ABC type efflux pump system that relies primarily on metabolic energy, energy that is derived from the richness and practically unlimited supply of carbohydrates continuously replenished by the digestive processes of the human host.

The demonstration of pH modulated efflux pump activity is extremely significant for the design of agents that are to serve as efflux pump inhibitors (EPIs). If the agent is to be an effective adjuvant to antibiotic therapy for the management of a food-borne infection caused by an efflux mediated MDR Gram negative bacterium such as an *E. coli* strain, it must be active against the ABC transporter that is protecting the bacterium from the toxic agents of the colon, and during antibiotic therapy. EPIs whose effectiveness is shown against RND type efflux pump systems may not be effective in the environment in which the offensive bacterium resides. Consequently, an EPI that is to serve as an adjunct to antibiotic therapy aimed at an efflux mediated MDR coliform infection should be evaluated for activity under physiological conditions, namely at a pH that favours the activity of ABC type efflux pump systems.

## Materials and Methods

### Materials

#### Media

Mueller-Hinton (MH) and Tryptic Soy in powder form for the preparation of broth and agar were purchased from Oxoid Ltd. (Basingstoke, UK). Phosphate buffered saline, glucose. tetracycline, EB, CCCP, PAβN and verapamil, purchased from Sigma-Aldrich Química SA (Madrid, Spain).

#### Bacteria

Wild-type *E. coli* K-12 AG100 strain (*argE3 thi-1 rpsL xyl mtl* delta (*gal-uvrB*) *supE44*) [24], was kindly offered by Hiroshi Nikaido, Department of Molecular and Cell Biology and Chemistry,University of California, Berkely, California, USA. This strain, sensitive to tetracycline (MIC of 2 mg/L) was exposed to increasing concentrations of tetracycline. The resulting strain AG100_TET_ is resistant to 8 mg/L tetracycline (MIC of 12 mg/L) and to other antibiotics of unrelated classes [5], [25]. This strain subsequently transferred to drug free medium or to medium containing 8 mg/L tetracycline and the EPI PAβN assumes initial susceptibility to tetracycline of 2 mg/L [5], [25].

### Methods

#### The effect of pH on the growth of *E. coli* AG100 and AG100_TET_

MH medium at pH 5, 7 and 8 (10 mL) was inoculated with 0.1 mL of an overnight culture and incubated at 37°C with shaking at 220 rpm until stationary phase. The OD of the culture at the end of incubation was divided by the number of hours for that culture to reach stationary phase. This calculation provides a “Growth Rate”, reflected by the slope of the growth curve: the steeper the slope (higher rate) the faster the growth.

#### Determination of minimum inhibitory concentrations (MICs)

MICs of PAβN, CCCP and verapamil against the strains employed in this study was conducted in MH medium adjusted to pH 5, 6, 7 and 8 by the broth microdilution method as per CLSI guidelines [5]. These MICs were performed for the purpose of selecting concentrations of these agents that have no significant effect on the growth of the strains used. The concentration of each agent employed did not exceed ¼ of its MIC.

#### Evaluation of efflux activity by a 96-well microplate screening method

The method that evaluates the ability of a bacterial strain to handle EB, that is, extrude EB, has been previously described [36] *via* an EB agar method. The principles of this method have been retained in a new assay that assesses the ability of a bacterial strain to extrude EB. This new assay employs a 96-well microplate in the manner employed for the determination of MICs. Briefly, varying concentrations of EB in TSB of varying pH were added to the wells after which time an inoculum of bacteria corresponding to a 0.5 McFarland was added. The plates were incubated for 16 hours and the lowest concentration of EB that promotes fluorescence of the contents of the well recorded and the plates photographed under UV light in a Gel-doc XR transiluminator (Bio-Rad, Hertfordshire, UK).

#### Assessment of putative efflux pump activity of *E. coli* AG100 and AG100_TET_ by a semi-automated fluorometric method

Detection of efflux pump activity in *E. coli* AG100 and AG100_TET_ was conducted by a semi-automated fluorometric method, previously described by us [34]. However, we have introduced a modification that affords the evaluation of efflux without the need to centrifuge for the removal of EB. This modification is described in the section that evaluates efflux of EB after addition of glucose or of agents that are being evaluated for activity against efflux of EB.

For the assessment of accumulation of EB and conditions that affect it the following was performed. Briefly, strains were cultured in MH broth medium until they reached an OD of 0.6 at 600 nm and aliquots of 1.0 mL were centrifuged twice at 13,000 rpm for 3 minutes. The pellets were re-suspended in saline and the OD adjusted to 0.6 in saline of pH 5, 7 and 8. Aliquots of 0.045 mL were transferred to microtubes of 0.2 mL and 0.045 mL of EB in glucose-free saline of pH 5, 7 and 8 added to the respective tubes (same pH). The final concentration of EB for all experiments was 1.0 mg/L. Concentrations of EB much greater than 1.0 mg/L exceed the ability of the cell to extrude the agent, the level of intracellular agent rapidly increases and results in its intercalation between the nucleic bases of DNA. EB when bound to DNA is no longer available for extrusion [25]. The tubes were transferred to the Rotor-Gene^TM^ 3000 thermocycler (Corbett Research, Sydney, Australia) programmed for 30–40 cyles of 1 minute (approximately 30–40 minutes) at a constant temperature of 37°C. Accumulation of EB of each tube was followed on a real-time basis by the assessment of fluorescence emitted. Excitation and emission wavelengths were 530 nm (bandpass) and 585 nm (highpass), respectively. Whereas the medium containing 1.0 mg/L of EB does not appreciably fluoresce, as the concentration of EB builds up in the periplasm of the Gram-negative bacterium, fluorescence is readily detected by the instrument [34].

The assessment of efflux of EB at pH 5, 7, and 8 was conducted as follows: accumulation of EB at pH 5, 7 and 8, as described above, was first conducted for up to 25 minutes, after which time the instrument was stopped and 0.010 mL of saline at pH 5, 7 and 8 lacking and containing glucose to yield a concentration of 0.4% was added to the respective tubes. The tubes were then transferred to the instrument and the instrument re-started. The total amount of time between this addition and the time the instrument was re-started did not exceed 2.5 minutes. Fluorescence was followed for a minimum period of 10 minutes. Data presented is for pH 5 and 8; at pH 6 and 7 data is intermediate between the ranges of pH and is not shown.

The effects of varying concentrations of CCCP, PAβN and verapamil on the efflux of EB was carried out as follows: accumulation of EB in glucose-free saline at pH 5, 7 and 8 was conducted as described above and when the plateau of accumulation was reached, the instrument was stopped and 0.045 ml of glucose-saline at pH 5, 7 and 8 containing varying concentrations of CCCP, PAβN and verapamil was added to the respective tubes. The tubes were placed into the instrument, the instrument re-started and fluorescence followed for up to 15 minutes. The total time for these additions did not exceed 2.5 minutes. For some experiments, the addition of CCCP at varying concentrations was in matched pH glucose-free medium. This component of the experiment afforded an additional control that would define any role of metabolic energy in conjunction with any effect produced by given concentrations of CCCP on efflux of EB and the modulation of efflux at a given pH.

## References

[1] Cole CB, Fuller R (1984). Bile acid deconjugation and attachment of chicken gut bacteria: their possible role in growth depression.. Br Poult Sci.

[2] Saier MH, Paulsen IT, Sliwinski MK, Pao SS, Skurray RA (1998). Evolutionary origins of multidrug and drug-specific efflux pumps in bacteria.. FASEB J.

[3] Nikaido H (1998). Multiple antibiotic resistance and efflux.. Curr Opin Microbiol.

[4] Li XZ, Nikaido H (2004). Efflux-mediated drug resistance in bacteria.. Drugs.

[5] Viveiros M, Jesus A, Brito M, Leandro C, Martins M (2005). Inducement and reversal of tetracycline resistance in *Escherichia coli* K-12 and expression of proton gradient-dependent multidrug efflux pump genes.. Antimicrob Agents Chemother.

[6] Nikaido H, Zgurskaya HI (2001). AcrAB and related multidrug efflux pumps of *Escherichia coli*.. J Mol Microbiol Biotechnol.

[7] Van Bambeke F, Balzi E, Tulkens PM (2000). Antibiotic efflux pumps.. Biochem Pharmacol.

[8] Pietras Z, Bavro VN, Furnham N, Pellegrini-Calace M, Milner-White EJ (2008). Structure and mechanism of drug efflux machinery in Gram negative bacteria.. Curr Drug Targets.

[9] Nikaido H (1996). Multidrug efflux pumps of gram-negative bacteria.. J Bacteriol.

[10] Elkins CA, Nikaido H (2003). 3D structure of AcrB: the archetypal multidrug efflux transporter of Escherichia coli likely captures substrates from periplasm.. Drug Resist Updat.

[11] Seeger MA, Diederichs K, Eicher T, Brandstätter L, Schiefner A (2008). The AcrB efflux pump: conformational cycling and peristalsis lead to multidrug resistance.. Curr Drug Targets.

[12] Ponte-Sucre A (2007). Availability and applications of ATP-binding cassette (ABC) transporter blockers.. Appl Microbiol Biotechnol.

[13] Turina P, Rebecchi A, D'Alessandro M, Anefors S, Melandri BA (2006). Modulation of proton pumping efficiency in bacterial ATP synthases.. Biochim Biophys Acta.

[14] Feniouk BA, Suzuki T, Yoshida M (2006). The role of subunit epsilon in the catalysis and regulation of FOF1-ATP synthase.. Biochim Biophys Acta.

[15] Davidson AL, Chen J (2004). ATP-binding cassette transporters in bacteria.. Annu Rev Biochem.

[16] Deckers-Hebestreit G, Greie J, Stalz W, Altendorf K (2000). The ATP synthase of *Escherichia coli*: structure and function of F(0) subunits.. Biochim Biophys Acta.

[17] Mulkidjanian AY, Heberle J, Cherepanov DA (2006). Protons at interfaces: Implications for biological energy conservation.. Biochim Biophys Acta.

[18] Michell P (1966). Chemiosmotic coupling in oxidative and photosynthetic phosphorylation.. Physiol Rev.

[19] Krulwich TA, Ito M, Gilmur R, Sturr MG, Guffanti AA, Hicks DB (1996). Energetic problems of extremely alkaliphilic aerobes.. Biochim Biophys Acta.

[20] Guffanti AA, Mann M, Sherman TL, Krulwich TA (1984). Patterns of electrochemical proton gradient formation by membrane vesicles from an obligatory acidophilic bacterium.. J Bacteriol.

[21] Cherepanov DA, Junge W, Mulkidjanian AY (2004). Proton transfer dynamics at the membrane/water interface: dependence on the fixed and mobile pH buffers, on the size and form of membrane particles, and on the interfacial potential barrier.. Biophys J.

[22] Gunn JS (2008). The *Salmonella* PmrAB regulon: lipopolysaccharide modifications, antimicrobial peptide resistance and more.. Trends Microbiol.

[23] Perez J, Groisman EA (2007). Acid pH activation of the PmrA/PmrB two component regulatory system of *Salmonella enterica*.. Mol Micorbiol.

[24] Tucker DL, Tucker N, Conway T (2002). Gene expression profiling of the pH response in *Escherichia coli*.. J Bacteriol.

[25] Viveiros M, Dupont M, Rodrigues L, Couto I, Davin-Regli A (2007). Antibiotic stress, genetic response and altered permeability of *E. coli*.. PLoS ONE.

[26] Lomovskaya O, Zgurskaya HI, Totrov M, Watkins WJ (2007). Waltzing transporters and ‘the dance macabre’ between humans and bacteria.. Nat Rev Drug Discov.

[27] Russell JB, Diez-Gonzalez F (1998). The effects of fermentation acids on bacterial growth.. Adv Microb Physiol.

[28] Su CC, Nikaido H, Yu EW (2007). Ligand-transporter interaction in the AcrB multidrug efflux pump determined by fluorescence polarization assay.. FEBS Lett.

[29] Thota N, Koul S, Reddy MV, Sangwan PL, Khan IA (2008). Citral derived amides as potent bacterial NorA efflux pump inhibitors.. Bioorg Med Chem.

[30] Rodrigues L, Wagner D, Viveiros M, Sampaio D, Couto I (2008). Thioridazine and chlorpromazine inhibition of ethidium bromide efflux in *Mycobacterium avium* and *Mycobacterium smegmatis*.. J Antimicrob Chemother.

[31] Hayes ET, Wilks JC, Sanfilippo P, Yohaness E, Tate DP (2006). Oxygen limitation modulates pH regulation of catabolism and hydrogenases, multi-drug transporters, and envelope composition in *Escherichia coli* K-12.. BMC Microbiol.

[32] Lee EW, Chen J, Huda MN, Kuroda T, Mizushima T (2003). Functional cloning and expression of *emeA*, and characterization of EmeA, a multidrug efflux pump from *Enterococcus faecalis*.. Biol Pharm Bull.

[33] Lee EW, Huda MN, Kuroda T, Mizushima T, Tsuchiya T (2003). EfrAB, an ABC multidrug efflux pump in *Enterococcus faecalis*.. Antimicrob Agents Chemother.

[34] Viveiros M, Martins A, Paxão L, Rodrigues L, Kern W (2008). Demonstration of intrinsic efflux activity of Gram-negative bacteria by an automated ethidium bromide method.. Int J of Antimicrob Agents.

[35] Simon J, van Spanning RJ, Richardson DJ (2008). The organization of proton motive and non-proton motive redox loops in prokaryotic respiratory systems.. Biochim Biophys Acta.

[36] Martins M, Santos B, Martins A, Viveiros M, Couto I (2006). An instrument-free method for the demonstration of efflux pump activity of bacteria.. In Vivo.

